# Self-citation pattern among world's top 2 % of the scientists

**DOI:** 10.1016/j.heliyon.2025.e42471

**Published:** 2025-02-05

**Authors:** Berun A. Abdalla, Ayman M. Mustafa, Fattah H. Fattah, Fahmi H. Kakamad, Sami S. Omar, Ameer M. Salih, Aso S. Muhialdeen, Jaafar Omer Ahmed, Rawa Bapir, Shvan H. Mohammed, Karokh K. Mohammed, Hiwa O. Baba, Sasan M. Ahmed, Shevan M. Mustafa, Kayhan A. Najar

**Affiliations:** a Smart Health Tower, Madam Mitterrand Street, Sulaymaniyah, Kurdistan, Iraq; b Kscien Organization, Hamdi Street, Azadi Mall, Sulaymaniyah, Kurdistan, Iraq; c College of Medicine, University of Sulaimani, Madam Mitterrand Street, Sulaymaniyah, Kurdistan, Iraq; d Rizgary Oncology Center, Peshawa Qazi Street, Erbil, Kurdistan, Iraq; e Civil Engineering Department, College of Engineering, University of Sulaimani, Sulaymaniyah, Kurdistan, Iraq; f Psychology Department, Faculty of Art, Soran University, Soran, Iraq; g Department of Urology, Sulaymaniyah Surgical Teaching Hospital, Sulaymaniyah, Kurdistan, Iraq

**Keywords:** Scientific publishing, Self-citation, Impact factor, Citation analysis

## Abstract

Although self-citation is a common practice among scholars, its impact and significance remain under scrutiny within the academic community. The current study aimed to provide a detailed analysis and ranking of the top 2 % scientists on self-citation, ranked by Stanford University researchers. Data extraction and organisation were performed between January and February 2024. Self-citation percentages were collected for authors belonging to 20 fields and 174 subfields using Excel spreadsheets. Entities were categorised into quartiles based on their self-citation percentages and rankings were assigned accordingly. Comparative analyses were conducted to assess the impact of self-citations on overall rankings. The study focused on data published by a group of researchers from Stanford University regarding the top 2 % of researchers, analysing the career-long records of 204,643 scholars and 210,198 scholars for the most recent year, 2022. Notably, in the single-year analysis, among the top 20 countries, approximately eight (40 %) exhibited self-citation percentages exceeding the average of 25.96 %. The self-citation percentages ranged from 4.47 % in Economics and Business to 20.88 % in Physics and Astronomy. Regarding career-long analysis, the percentage of self-citations ranged from 22.84 % in Poland to 41.31 % in Armenia, with significant drop in rankings among most entities when self-citations were excluded. These findings highlight the dramatic impact of self-citation exclusion on the rankings of the top 2 % of the researchers, underscoring the critical importance of accounting for self-citation in ranking assessments.

## Highlights

• Self-citation is a common practice among scholars worldwide.
• Citation metrics are vital for equitable scientific assessments.
• We analysed self-citation in top 2 % scientists.
• Excluding self-citation impacted rankings of countries, institutes, fields, subfields.

## 1 Introduction

Research productivity is crucial to enhance the visibility of researchers, institutions, and countries. The quality of a researcher's work is often assessed based on the number of citations their scientific publications receive. At both the meso and macro levels, institutional and national rankings rely on various citation-based bibliometric indicators [1]. Self-citations can affect the total citation count, potentially distorting these indicators and affecting their accuracy and measurements. Although self-citation is a significant factor in evaluating researchers' impact, rankings, and other quality metrics, it can present challenges in accurately assessing the scientific output of authors, institutions, and countries [2,3].

In particular, self-citation has emerged as a contentious topic in scholarly circles. Self-citation refers to the practice where authors cite their own previous publications in their current research. Specifically, it occurs when both the citing and cited publications share at least one author [4]. Various perspectives on self-citations have been explored, including author self-citations, journal self-citations, institutional self-citations, country self-citations, discipline self-citations, and even webpage self-citations [5].

### 1.1 Literature review

Acceptability of the self-citation percentage can vary depending on the field of study, nature of the research, and cultural norms within the academic community. Generally, a moderate level of self-citation, typically ranging from 10 % to 20 %, is considered acceptable and even expected in many disciplines, especially the natural sciences and medicine [6]. This level of self-citation is often viewed as a natural part of academic discourse, especially when authors are referencing their previous work that is directly relevant to the current research. However, this range can be affected by several factors. In some disciplines, especially those with highly specialized research or a relatively small number of researchers, higher self-citation rates may be more common and acceptable. Conversely, exceeding this range, particularly surpassing 20 %, may raise concerns about potential biases or attempts to artificially inflate citation metrics for personal gain. Excessive self-citation may give the impression of self-promotion or an effort to manipulate citation-based evaluations, rather than being based on genuine merit [7].

Critical findings highlight that excessive self-citation can artificially inflate impact factors, with studies revealing that self-citations can cause a 54 % increase or 42 % decrease in journal Impact factors [8]. Despite these insights, significant research gaps persist, including limited semantic analysis of self-citation detection, and insufficient cross-disciplinary comparative studies. Researchers at Stanford University have developed a database of top-cited scientists by employing standardised metrics, such as citations, h-index, and composite c-score from Scopus data, updated until 2022 [9]. This database offers insights into scientists’ careers, categorising them across 22 fields and 174 subfields. It includes the top 100,000 scientists by c-score, or those ranking above the 2nd percentile in their subfields. Although the database is valuable for promoting accurate Scopus profiles, it lacks mechanisms to assess self-citations, underscoring the need to study self-citation rates and their impact on metrics. This study aims to utilise this database to assess and rank countries, institutes, fields, and subfields based on their self-citation behaviours, providing insights into the frequency and consequences of self-citation among the top 2 % of scientists worldwide.

## 2 Methods

### 2.1 Data extraction and organisation

**Fig. 1:**
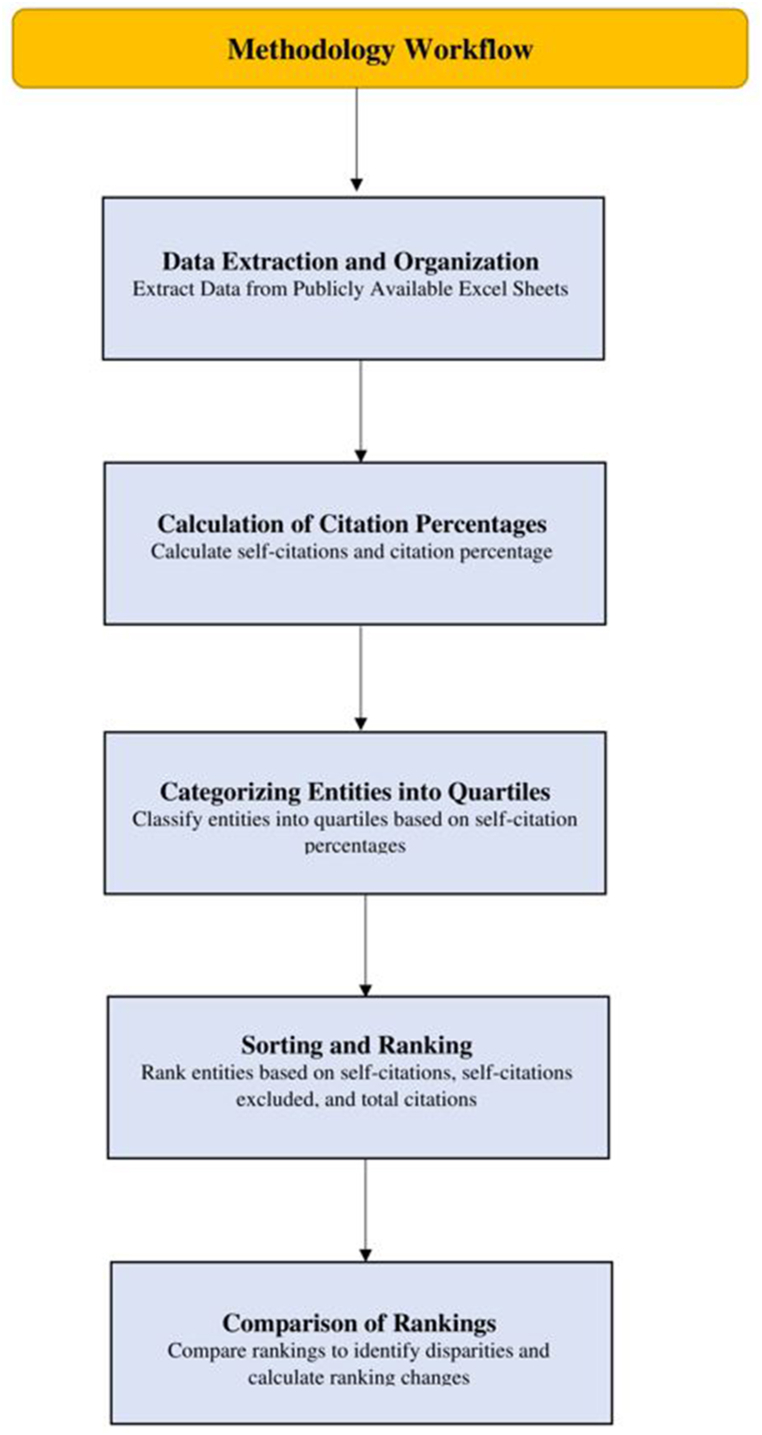
Methodological flow diagram.

This study was conducted between January and February 2024. Ethical approval was not required, because this study used data obtained from openly accessible sources. The data were extracted from Excel sheets published by Ioannidis on 1 October 2023 [9]. The dataset encompasses citation data for a broad range of 20 fields and 174 subfields, covering both career-long (1996–2022) and single recent year (2022) periods. The data, initially at the individual scholar level, were aggregated to the country, field, subfield, and institutional levels using Excel. Self-citations were computed retrospectively using an author-based approach, considering all citations referencing previous works by the same author within the study period. For country-level aggregation, the self-citations and total citations of individual scholars were summed by country to obtain country-specific citation metrics. At the field and subfield levels, citations were aggregated and summed for each field and subfield to determine field- and subfield-specific citation metrics. For institutional aggregation, the citations were similarly summed for scholars affiliated with each institution, yielding institution-specific citation metrics (Fig. 1).

### 2.2 Calculation of citation percentages

For each entity (country, field, subfield, and institution), the percentages of self-citations and those without them were calculated using the following formula in a separate Excel spreadsheet:

Percentageofself−citations=(Self−citations/totalcitations)×100

Percentageofcitations(excludingself−citations)=(Citationsexcludingself−citations/Totalcitations)×100

### 2.3 Categorising entities into quartiles

The entities were categorised into quartiles based on their self-citation percentages. Excel's quartile function was used to divide the entities into four quartiles based on their self-citation percentages: Quartile 1: Percentages of self-citations between 75 and 100; Quartile 2: Percentages of self-citations between 50 and 75; Quartile 3 (Q3): Percentages of self-citations between 25 and 50; and Quartile 4 (Q4): Percentages of self-citation between 0 and 25 Each entity was assigned a quartile label (Q1, Q2, Q3, or Q4) based on its self-citation percentage. Additionally, countries were categorised as low-income, lower-middle income, upper-middle income, or high-income based on the World Bank classification [10].

### 2.4 Sorting and ranking

Each sheet was sorted in descending order based on the calculated percentages of self-citations, citations excluding self-citations, and total citations for each entity. Rankings were then assigned to each entity based on their sorted order. Three distinct ranking methods were used in this study. First, the total ranking was determined based on the total citation count. Second, ranking was established using the percentage of citations, excluding self-citations. Finally, a separate ranking was derived based on the percentage of self-citations.

### 2.5 Comparison of rankings

The rankings derived from the self-citation percentage, self-citation-excluded ranking, and total citation ranking for each entity were compared. Excel functions were used to identify any disparities or similarities between the rankings. Differences in rankings were analysed to understand the impact of self-citation on the overall ranking of entities (Fig. 1). The change in ranking of each entity was calculated using the following formula:

Ranking change = Ranking based on total citations - Ranking based on self-excluded citations

## 3 Results

In this section, the findings of the analysis on self-citations and their impact on citation rankings across various categories are presented. The results are divided into two primary categories: 3.1 Single-year analysis and 3.2 Career-long data analysis. Each category will be discussed separately, with a focus on the ranking shifts observed at the country, field, sub-field, and institutional levels.

### 3.1 Single-year analysis

In this section, a total of 210,198 authors were analysed for their publication records in 2022, with the analysis categorised by country, field, sub-field, and institution.

#### 3.1.1 Country ranking analysis

The average self-citation percentage of the top 20 countries was approximately 25.96 %. Of these, eight (40 %) had self-citation percentages above this average. Notably, six of the top 10 (60 %) countries with the highest self-citations were either low- or lower-middle-income countries, highlighting the potential role of regional publication practices and local research ecosystems. Armenia ranked the highest in terms of self-citations. It was ranked 92nd in terms of total citations, with 16,682 citations, but fell to the lowest rank when self-citations were excluded. This stark contrast in rankings underscores the influence of self-citations on national research metrics. Most countries experienced a decline in ranking when self-citations were excluded, with Egypt showing the most significant drop, from 38th to 156th place (Table .1, Suppl.1).

**Table 1:** Ranking of countries based on self-citations, total citations, and self-citations excluded, for 2022.

Country	Total-Citations	Rank	Self-Citation Excluded	%Self-Citation Excluded	Rank	Self-Citation	%Self-Citation	Rank	Quartile	Income Level	Ranking change
Armenia	16682	92	10128	60.71	166	6554	39.29	1	Q3	UMIC	−74
Afghanistan	645	149	429	66.51	165	216	33.49	2	Q3	LIC	−16
Ukraine	36354	80	25074	68.97	164	11280	31.03	3	Q3	LMIC	−84
Uzbekistan	8107	102	5609	69.19	163	2498	30.81	4	Q3	LMIC	−61
French Guiana	900	144	631	70.11	162	269	29.89	5	Q3	HIC	−18
Yemen	3064	122	2184	71.28	161	880	28.72	6	Q3	LIC	−39
New Caledonia	2358	129	1728	73.28	160	630	26.72	7	Q3	HIC	−31
Sudan	1928	135	1420	73.65	159	508	26.35	8	Q3	LIC	−24
Iraq	103645	58	77293	74.57	158	26352	25.43	9	Q3	UMIC	−100
Guinea-Bissau	2553	127	1937	75.87	157	616	24.13	10	Q4	LIC	−30
Egypt	521962	38	397079	76.07	156	124883	23.93	11	Q4	LMIC	−118
North Korea	424	154	324	76.42	155	100	23.58	12	Q4	LIC	−1
Russia	681435	37	520994	76.45	154	160441	23.55	13	Q4	UMIC	−117
Bosnia and Herzegovina	3264	120	2500	76.59	153	764	23.41	14	Q4	UMIC	−33
Madagascar	309	157	237	76.70	152	72	23.30	15	Q4	LIC	5
Burkina Faso	4190	114	3266	77.95	151	924	22.05	16	Q4	LIC	−37
Algeria	29043	84	22751	78.34	150	6292	21.66	17	Q4	LMIC	−66
Latvia	10317	96	8132	78.82	149	2185	21.18	18	Q4	HIC	−53
Morocco	64751	67	51108	78.93	148	13643	21.07	19	Q4	LMIC	−81
Mali	2616	126	2103	80.39	147	513	19.61	20	Q4	LIC	−21

UMIC: upper middle-income country, LIC: low-income country, HIC: high income country, LMIC: low middle-income country.

#### 3.1.2 Field ranking analysis

Self-citation rates across the 20 major academic fields ranged from 4.47 % in Economics and Business to 20.88 % in Physics and Astronomy. Excluding self-citations from total citations caused significant shift in field ranking. For instance, Clinical Medicine, which ranked first with 96,021,512 citations, dropped to 12th when self-citations were excluded, although 10.69 % of the citations in this field were self-citations. Most fields fell in rank when self-citations were removed, with Physics and Astronomy experiencing the largest drop from the 4th to 20th place, a 16-place decline, reflecting the substantial role that self-citations play in fields with high research output and global impact (Table 2).

**Table 2:** Ranking of major fields based on self-citations, total citations, and self-citations excluded, for 2022.

Field	Total-Citations	Rank	Self-Citation excluded	% Self-Citation excluded	Rank	Self-citation	%Self -Citation	Quartile	Rank	Ranking change
Physics & Astronomy	19597783	4	15505967	79.12	20	4091816	20.88	Q4	1	−16
Historical Studies	204696	18	175674	85.82	19	29022	14.18	Q4	2	−1
Earth & Environmental Sciences	8201193	8	7182813	87.58	18	1018380	12.42	Q4	3	−10
Engineering	10871428	7	9586841	88.18	17	1284587	11.82	Q4	4	−10
Agriculture, Fisheries & Forestry	4630449	10	4085988	88.24	16	544461	11.76	Q4	5	−6
Biology	7777876	9	6875228	88.39	15	902648	11.61	Q4	6	−6
Mathematics & Statistics	1319287	15	1166981	88.46	14	152306	11.54	Q4	7	1
Chemistry	12945303	5	11543583	89.17	13	1401720	10.83	Q4	8	−8
Clinical Medicine	96021512	1	85758472	89.31	12	10263040	10.69	Q4	9	−11
Enabling & Strategic Technologies	20573912	3	18496734	89.90	11	2077178	10.10	Q4	10	−8
Built Environment & Design	873147	16	788407	90.29	10	84740	9.71	Q4	11	6
Biomedical Research	20902551	2	18974316	90.78	9	1928235	9.22	Q4	12	−7
Public Health & Health Services	2955122	13	2695209	91.20	8	259913	8.80	Q4	13	5
Psychology & Cognitive Sciences	3656143	12	3376530	92.35	7	279613	7.65	Q4	14	5
Information & Communication Technologies	12814677	6	11860836	92.56	6	953841	7.44	Q4	15	0
Visual & Performing Arts	5296	20	4905	92.62	5	391	7.38	Q4	16	15
Philosophy & Theology	85920	19	80574	93.78	4	5346	6.22	Q4	17	15
Social Sciences	2070022	14	1950965	94.25	3	119057	5.75	Q4	18	11
Communication & Textual Studies	308115	17	291725	94.68	2	16390	5.32	Q4	19	15
Economics & Business	3859622	11	3687076	95.53	1	172546	4.47	Q4	20	10

#### 3.1.3 Sub-field ranking analysis

Among the 174 major subfields analysed, Nuclear and Particle Physics ranked 9th in total citations with 5,836,007 but fell to 174th when self-citations were excluded, indicating that 33.40 % of its citations were self-citations. Most subfields dropped in rank when self-citations were removed, with Nuclear and Particle Physics showing the largest decline, falling from 9th to 174th place, a 165-place drop, this marked decline illustrates the prominence of self-citing practices within subfields of specialized scientific research, where smaller, more insular research communities may heavily cite their own work. This highlights the need for careful interpretation of citation metrics in these subfields, as excessive self-citations may distort the true impact of research (Table 3, Suppl. 2).

**Table 3:** Ranking of subfields based on self-citations, total citations, and self-citations excluded, for 2022.

Subfield	Total-Citations	rank	Citations-self excluded	% self-excluded	rank	Self-Citations	%self-citation	Rank	Quartile	Ranking change
Nuclear & Particle Physics	5836007	9	3887041	66.60	174	1,948,966	33.40	1	Q3	−165
Astronomy & Astrophysics	2732948	23	1943387	71.11	173	789,561	28.89	2	Q3	−150
Zoology	114629	135	87097	75.98	172	27,532	24.02	3	Q4	−37
Mycology & Parasitology	454038	88	359357	79.15	171	94,681	20.85	4	Q4	−83
Archaeology	124356	133	102082	82.09	170	22,274	17.91	5	Q4	−37
Paleontology	346043	102	286469	82.78	169	59,574	17.22	6	Q4	−67
Aerospace & Aeronautics	368,733	100	306,449	83.11	168	62,284	16.89	7	Q4	−68
Allergy	566490	81	471455	83.22	167	95,035	16.78	8	Q4	−86
Ornithology	44762	149	37255	83.23	166	7507	16.77	9	Q4	−17
Optoelectronics & Photonics	1,285,068	50	1,075,761	83.71	165	209,307	16.29	10	Q4	−114
Applied Mathematics	334119	103	280860	84.06	164	53,259	15.94	11	Q4	−61
Forestry	327,403	104	276,145	84.34	163	51,258	15.66	12	Q4	−59
Anatomy & Morphology	42864	152	36279	84.64	162	6585	15.36	13	Q4	−10
Fisheries	484,104	86	409,842	84.66	161	74,262	15.34	14	Q4	−75
Microscopy	45853	147	38844	84.71	160	7009	15.29	15	Q4	−13
Civil Engineering	708,163	77	601,307	84.91	159	106,856	15.09	16	Q4	−82
Geology	165867	125	141716	85.44	158	24,151	14.56	17	Q4	−33
General Mathematics	428355	90	367537	85.80	157	60,818	14.20	18	Q4	−67
Entomology	377275	96	323839	85.84	156	53,436	14.16	19	Q4	−60
Legal & Forensic Medicine	173201	124	148673	85.84	155	24,528	14.16	20	Q4	−31

#### 3.1.4 Institute ranking analysis

Among 21,811 institutes analysed, the top 20 based on self-citations were examined. For example, Altai State University was ranked 15,323rd in total citations but dropped to 21,801th when self-citations were excluded. This university ranked 11th in self-citations, accounting for 85.37 % of its total citations. Most institutes experienced a decrease in rank when self-citations were removed, highlighting the role of institutional self-citation practices in artificially inflating rankings. The Kumaraguru College of Technology had the most significant decline, falling from 11,479th to 21,809th place, a drop of 10,330 positions (Table 4, Suppl. 3).

**Table 4:** Ranking of institutes based on self-citations, total citations, and self-citations excluded, for 2022.

Institute	country	Total-citation	rank	Self-citation excluded	%Self-Citation excluded	rank	Self-Citation	%Self-citation	rank	Quartile	Ranking Change
ASTRAX, Inc.	Japan	81	21542	0	0	21811	81	100	1	Q1	−269
Université Oran 1	Algeria	103	21263	3	2.91	21810	100	97.09	2	Q1	−547
Kumaraguru College of Technology	India	976	11479	42	4.30	21809	934	95.70	3	Q1	−10330
Suqian University	China	142	20738	7	4.93	21808	135	95.07	4	Q1	−1070
Institute of Natural Sciences and Applied Technology	India	658	13618	36	5.47	21807	622	94.53	5	Q1	−8189
Kherson State University	Ukraine	404	16441	34	8.42	21806	370	91.58	6	Q1	−5365
Belgorod State Technological University named after V.G.Shoukhov	Russia	219	19426	20	9.13	21805	199	90.87	7	Q1	−2379
Computing Center FEB RAS	Russia	149	20623	17	11.41	21804	132	88.59	8	Q1	−1181
Satyamjayatu: The Science & Ethics Foundation	India	302	18013	35	11.59	21803	267	88.41	9	Q1	−3790
Surgut State University	Russia	133	20860	19	14.29	21802	114	85.71	10	Q1	−942
Altai State University, Barnaul	Russia	492	15323	72	14.63	21801	420	85.37	11	Q1	−6478
V.I. Vernadsky Crimean Federal University	Russia	156	20528	23	14.74	21800	133	85.26	12	Q1	−1272
Polzunov Altai State Technical University	Russia	125	20982	19	15.2	21799	106	84.8	13	Q1	−817
Governors State University	USA	295	18114	46	15.59	21798	249	84.41	14	Q1	−3684
Nikola Vaptsarov Naval Academy	Bulgaria	124	20997	21	16.94	21797	103	83.06	15	Q1	−800
Rissho University	Japan	304	17977	55	18.09	21796	249	81.91	16	Q1	−3819
I.I. Schmalhausen Institute of Zoology of National Academy of Sciences of Ukraine	Ukraine	524	14983	95	18.13	21795	429	81.87	17	Q1	−6812
Oli Health Magazine Organization	Rwanda	382	16769	74	19.37	21794	308	80.63	18	Q1	−5025
Institute for Chemistry, Technology and Metallurgy	Serbia	198	19782	39	19.70	21793	159	80.30	19	Q1	−2011
Institute of High Temperature Electrochemistry of the Ural Branch of the Russian Academy of Sciences	Russia	205	19663	41	20	21792	164	80	20	Q1	−2129

### 3.2 Career-long data analysis

In this section, a total of 204,643 authors were studied for their career-long publication (1996–2022) records, with the analysis categorised by country, field, sub-field, and institution.

#### 3.2.1 Country ranking analysis

The self-citation percentages ranged from 22.84 % in Poland to 41.31 % in Armenia. As observed in the single-year analysis, excluding self-citations caused significant changes in rankings. For instance, Ukraine dropped from 64th to 166th place when self-citations were removed, despite being ranked 3rd in self-citations. Most of the top 20 countries (75 %) were in Q3, with 60 % being in the upper or lower-middle income group. Many countries experienced a drop in rank when self-citations were excluded, with Russia experiencing the largest decline, falling from 31st to 160th place (Table .5, Suppl. 4). These further emphasize the need to account for self-citations in evaluating global research impact.

**Table 5:** Ranking of countries based on self-citations, total citations, and self-citations excluded, from 1996 to 2022.

Country	Total-Citation	Rank	Self-Citation excluded	% self-Citation excluded	Rank	Self-Citation	%Self-Citation	Rank	Quartile	Income level	Ranking change
Armenia	129769	82	76164	58.69	168	53605	41.31	1	Q3	UMIC	−86
Greenland	14438	131	9680	67.05	167	4758	32.95	2	Q3	HIC	−36
Ukraine	331474	64	228074	68.81	166	103400	31.19	3	Q3	LMIC	−102
Guinea-Bissau	25573	116	17807	69.63	165	7766	30.37	4	Q3	LIC	−49
Bosnia and Herzegovina	17814	125	12545	70.42	164	5269	29.58	5	Q3	UMIC	−39
Benin	6255	145	4471	71.48	163	1784	28.52	6	Q3	LMIC	−18
Eswatini	3485	156	2496	71.62	162	989	28.38	7	Q3	LMIC	−6
Montenegro	15381	128	11083	72.06	161	4298	27.94	8	Q3	UMIC	−33
Russia	6037446	31	4356964	72.17	160	1680482	27.83	9	Q3	UMIC	−129
Iraq	132813	80	97011	73.04	159	35802	26.96	10	Q3	UMIC	−79
Latvia	65956	94	48350	73.31	158	17606	26.69	11	Q3	HIC	−64
San Marino	3359	157	2467	73.44	157	892	26.56	12	Q3	HIC	0
Algeria	95304	90	70319	73.78	156	24985	26.22	13	Q3	LMIC	−66
Romania	916711	48	682781	74.48	155	233930	25.52	14	Q3	HIC	−107
Sierra Leone	8154	141	6083	74.60	154	2071	25.40	15	Q3	LIC	−13
Côte d'Ivoire	4539	149	3447	75.94	153	1092	24.06	16	Q4	LMIC	−4
North Macedonia	26087	114	19967	76.54	152	6120	23.46	17	Q4	UMIC	−38
Senegal	33222	107	25572	76.97	151	7650	23.03	18	Q4	LMIC	−44
Falkland Islands	4030	152	3105	77.05	150	925	22.95	19	Q4	NA	2
Poland	6509489	29	5022485	77.16	149	1487004	22.84	20	Q4	HIC	−120

UMIC: upper middle-income country, LIC: low-income country, HIC: high income country, LMIC: low middle-income country.

#### 3.2.2 Field ranking analysis

The effect of self-citation on rankings has been examined in various academic fields. When self-citations were excluded, Physics and Astronomy dropped from 3rd to 20th place, with self-citations comprising 20.06 % of its total. Economics and Business had the lowest self-citation percentage of 5.20 %. All fields were in the fourth quartile of self-citation counts. Most fields experienced a decline in ranking when self-citations were removed, with Physics and Astronomy showing the most significant drop (Table 6).

**Table 6:** Ranking of major fields based on self-citations, total citations, and self-citations excluded, from 1996 to 2022.

Field	Total-Citations	Rank	Self-Citation excluded	% Self-citation excluded	Rank	Self-Citation	%Self-Citation	Rank	Quartile	Ranking change
Physics & Astronomy	210,949,287	3	168,638,853	79.94	20	42,310,434	20.06	1	Q4	−17
Earth & Environmental Sciences	64,786,543	9	54,869,844	84.69	19	9,916,699	15.31	2	Q4	−12
Engineering	82,585,133	7	70,523,674	85.40	18	12,061,459	14.60	3	Q4	−11
Agriculture, Fisheries & Forestry	35,536,277	11	30,429,869	85.63	17	5,106,408	14.37	4	Q4	−6
Chemistry	128,535,280	5	110,127,085	85.68	16	18,408,195	14.32	5	Q4	−11
Biology	72,634,825	8	62,842,177	86.52	15	9,792,648	13.48	6	Q4	−7
Historical Studies	2,260,178	18	1,970,295	87.17	14	289,883	12.83	7	Q4	4
Enabling & Strategic Technologies	131,541,420	4	114,709,042	87.20	13	16,832,378	12.80	8	Q4	−9
Mathematics & Statistics	13,232,256	15	11,544,833	87.25	12	1,687,423	12.75	9	Q4	3
Built Environment & Design	4,834,093	16	4,242,066	87.75	11	592,027	12.25	10	Q4	5
Biomedical Research	230,097,694	2	202,407,997	87.97	10	27,689,697	12.03	11	Q4	−8
Clinical Medicine	897,462,195	1	789,843,912	88	9	107,618,283	12	12	Q4	−8
Public Health & Health Services	26,867,239	12	23,904,866	88.97	8	2,962,373	11.03	13	Q4	4
Information & Communication Technologies	89,248,695	6	80,487,513	90.18	7	8,761,182	9.82	14	Q4	−1
Psychology & Cognitive Sciences	37,516,480	10	33,861,222	90.26	6	3,655,258	9.74	15	Q4	4
Visual & Performing Arts	55,275	20	50,804	91.91	5	4471	8.09	16	Q4	15
Social Sciences	17,501,408	14	16,233,855	92.76	4	1,267,553	7.24	17	Q4	10
Philosophy & Theology	866,910	19	804,308	92.78	3	62,602	7.22	18	Q4	16
Communication & Textual Studies	2,306,845	17	2,151,926	93.28	2	154,919	6.72	19	Q4	15
Economics & Business	25,160,799	13	23,853,425	94.80	1	1,307,374	5.20	20	Q4	12

#### 3.2.3 Sub-field ranking analysis

Among the 174 subfields, Nuclear and Particle Physics exhibited the largest decline, falling from 11th to 174th place when self-citations were excluded, a dramatic 163-place drop. Astronomy and astrophysics dropped from 20th to 173rd, with self-citations accounting for 27.94 % of its total. These findings highlight the importance of understanding the dynamics of self-citation in specialized academic areas, where internal citation practices can greatly affect the perceived impact of research. Regarding the quartiles of these subfields, the majority of them belonged to Q4, except for Nuclear and Particle Physics, Astronomy and Astrophysics which belonged to Q3 (Table 7, Suppl. 5).

**Table 7:** Ranking of subfields based on self-citations, total citations, and self-citations excluded, from 1996 to 2022.

Subfield	Total citations	Rank	Self-Citation excluded	%Self-Citation excluded	Rank	Self-citations	% Self-citations	ranks	Quartile	Ranking change
Nuclear & Particle Physics	49566227	11	35060599	70.73	174	14505628	29.27	1	Q3	−163
Astronomy & Astrophysics	29634350	20	21355049	72.06	173	8279301	27.94	2	Q3	−153
Zoology	1028845	133	786728	76.47	172	242117	23.53	3	Q4	−39
Mycology & Parasitology	3921752	93	3044485	77.63	171	877267	22.37	4	Q4	−78
Optoelectronics & Photonics	13945222	42	11225797	80.50	170	2719425	19.50	5	Q4	−128
Aerospace & Aeronautics	3372267	100	2718733	80.62	169	653534	19.38	6	Q4	−69
Optics	10903368	52	8891996	81.55	168	2011372	18.45	7	Q4	−116
Civil Engineering	4089420	91	3355451	82.05	167	733969	17.95	8	Q4	−76
Entomology	3772438	94	3095513	82.06	166	676925	17.94	9	Q4	−72
Paleontology	4141636	90	3411782	82.38	165	729854	17.62	10	Q4	−75
Allergy	5818247	78	4808558	82.65	164	1009689	17.35	11	Q4	−86
Ornithology	558637	146	463073	82.89	163	95564	17.11	12	Q4	−17
Meteorology & Atmospheric Sciences	22436970	25	18606221	82.93	162	3830749	17.07	13	Q4	−137
Forestry	2496568	108	2071999	82.99	161	424569	17.01	14	Q4	−53
Inorganic & Nuclear Chemistry	14542462	39	12088024	83.12	160	2454438	16.88	15	Q4	−121
Tropical Medicine	5945082	77	4953063	83.31	159	992019	16.69	16	Q4	−82
Fisheries	3993021	92	3331595	83.44	158	661426	16.56	17	Q4	−66
Archaeology	1123495	128	939536	83.63	157	183959	16.37	18	Q4	−29
Applied Mathematics	2948420	104	2470667	83.80	156	477753	16.20	19	Q4	−52
Mechanical Engineering & Transports	12633879	46	10592471	83.84	155	2041408	16.16	20	Q4	−109

#### 3.2.4 Institute ranking analysis

Among the 24,394 institutes studied, the top 20 for self-citations were highlighted. The Technocrat Society had the highest number of self-citations, accounting for 93.26 % of its total. Most institutes experienced a ranking drop when self-citations were excluded. The Vel Tech Rangarajan Dr. Sagunthala R&D Institute had the largest drop, falling from the 6440th to 24383rd place, a 17,943-position decline (Table 8, Suppl. 6).

**Table 8:** Ranking of institutes based on self-citations, total citations, and self-citations excluded, from 1996 to 2022.

Institute	Country	Total-citations	Rank	Self-citation excluded	%Self-citation excluded	Rank	Self-Citations	%Self-Citations	Rank	Quartile	Ranking change
Technocrat Society	India	1158	23324	78	6.74	24394	1080	93.26	1	Q1	−1070
Muş Alparslan Üniversitesi	Turkey	1709	22120	199	11.64424	24393	1510	88.35576	2	Q1	−2273
Shonan Institute of Chemoinformatics and Mathematical Chemistry	Japan	2156	21118	256	11.87384	24392	1900	88.12616	3	Q1	−3274
University of Chemistry and Technology	Czech Republic	5257	15587	778	14.79932	24391	4479	85.20068	4	Q1	−8804
Tropical Entomology Research Center	Italy	1454	22699	242	16.64374	24390	1212	83.35626	5	Q1	−1691
Rissho University	Japan	3843	17708	742	19.30783	24389	3101	80.69217	6	Q1	−6681
University of Penza	Russia	731	24092	150	20.51984	24388	581	79.48016	7	Q1	−296
Institute of Physics and Technology	Russia	2126	21192	458	21.5428	24387	1668	78.4572	8	Q1	−3195
National Aviation University	Ukraine	1215	23196	264	21.7284	24386	951	78.2716	9	Q1	−1190
Brilliant Light Power, Inc.	USA	2076	21309	455	21.91715	24385	1621	78.08285	10	Q1	−3076
Alexandria Higher Institute of Engineering and Technology	Egypt	2246	20899	496	22.0837	24384	1750	77.9163	11	Q1	−3485
Vel Tech Rangarajan Dr.Sagunthala R&D Institute of Science and Technology	India	26457	6440	6069	22.93911	24383	20388	77.06089	12	Q1	−17943
Strategic Solutions Technology Group	Israel	570	24267	146	25.61404	24382	424	74.38596	13	Q2	−115
Guangdong University of Education	China	2807	19678	767	27.32455	24381	2040	72.67545	14	Q2	−4703
Universiti Pendidikan Sultan Idris	Malaysia	2966	19328	820	27.64666	24380	2146	72.35334	15	Q2	−5052
Quantum Age	USA	507	24312	143	28.21	24379	364	71.79	16	Q2	−67
Institute of Engineering and Management	India	5148	15720	1475	28.6519	24378	3673	71.3481	17	Q2	−8658
Velagapudi Ramakrishna Siddhartha Engineering College	India	844	23916	247	29.2654	24377	597	70.7346	18	Q2	−461
Government College for Women	India	1728	22078	508	29.39815	24376	1220	70.60185	19	Q2	−2298
Joint Directorate of the Mordovia State Nature Reserve and National Park “Smolny”	Russia	938	23759	285	30.3838	24375	653	69.6162	20	Q2	−616

## 4 Discussion

The significance of self-citation cannot be overstated, particularly its role in correcting and preventing distortions in scholarly impact assessment [11]. Self-citation which is typically described as a citation in which both the citing and cited papers share at least one author can arise from the cumulative nature of individual research and personal satisfaction, or as a strategic tool to enhance visibility and authority within the scientific community. However, the practice of self-citation presents considerable challenges in accurately evaluating the impact and influence of scholarly work [12,13]. Analysis of self-citation percentages among countries underscores the global prevalence of this phenomenon. From 1996 to 2008, the rate of self-citation by countries has steadily increased, showing significant variations among countries and disciplines as well as depending on the number of authors involved [1]. A bibliometric study evaluating the scientific output of 238 countries from 1996 to 2017found a self-citation rate of 30.24 % [6]. Another investigation sourcing secondary data from the SCImago Journal and Country Ranking website reported a self-citation rate of 34.45 % [10]. Additionally, a study focusing on the 50 countries with the highest total number of citations in clinical neurology revealed a self-citation rate of 13.70 % [14]. When examining the impact of self-citation on the world's top 10 most research-productive countries, a self-citation rate of approximately 28.03 % was observed [1]. In the current study, which analysed self-citation rates among countries for the year 2022 and the career-long period from 1996 to 2022, the self-citation rates were approximately 16.10 % and 13.60 %, respectively. Notably, lower and middle-income countries exhibited higher rates of self-citations, highlighting the potential influence of contextual factors such as resource limitations or publishing norms on self-citation behaviours. This study's comparison of total citations, including and excluding self-citations, revealed significant fluctuations in the country rankings. This substantial variation in rankings, whether self-citations were included or excluded, is a common finding among the countries examined in this study, a phenomenon scarcely documented in the genuine literature [15]. Previous studies have revealed similar trends. For instance, a study of the top ten most research-productive countries found that China and Japan were the only nations showing a decline in variation when self-citations were excluded [1]. Another study reported minimal declines in country-ranking variations when self-citations were excluded [14]. Moreover, recent research on country-level self-citations has identified anomalous trends in certain countries, such as Italy, driven by citation-metrics-centered science policies. The study found that while country self-citations generally decreased over time, scholars in 12 out of 50 countries, including Egypt, Colombia, Indonesia, Malaysia, Pakistan, Romania, Iran, Italy, Saudi Arabia, Thailand, and Ukraine, exhibited unusual trends of excessive self-citation [16]. The observed increase in self-citation rates in these countries is linked to stringent scientific policies that promote high citation metrics. A prior study by the same researchers, published in 2019, examined Italy's self-citation practices and identified a connection to a 2010 policy that mandated specific productivity benchmarks for academic promotion, including publication volume and citation metrics. This citation-centric policy framework is believed to contribute to the atypical self-citation patterns observed in these countries [17]. These findings underscore the importance of considering self-citation practices in the assessment of research productivity and ranking at the national level.

The analysis of self-citation patterns across diverse academic fields and subfields provides valuable insights into the variations in scholarly practices across disciplines. These observations shed light on the differences in the research culture and norms prevalent in different fields. For instance, a study conducted in Saudi Arabia covering the period from 1996 to 2019 examined 146 medical specialties and revealed a self-citation rate of 11.19 % within these fields. Interestingly, a minimal decline in ranking was observed when self-citations were excluded from the total citations [18]. This study examined how often authors cite papers from their own country compared to the global presence, using the over-citation ratio (OCR). It was found that the OCR decreased from 1980 to 2010, particularly in fields of international interest such as astronomy and cancer, while it was higher in more nationally focused fields such as chemistry. The study also found that basic cancer research has a slightly higher OCR than clinical research, which helps contextualise whether certain citations are unusually nationalistic or typical.

Citation metrics are vital for equitable scientific assessments; however, addressing transparency issues, especially those concerning self-citations, is urgent. Kacem et al. used Clarivate Analytics Web of Science data to examine self-referencing across 15 disciplines, analysing 385,616 authors and 3,240,973 publications with 90,806,462 citations, 5 % of which were self-citations. Their study advocates the transparent tracking of self-citations to accurately understand citation behaviours and avoid misleading impact assessments. They proposed methodologies that incorporated all citation data, considering self-citations, collaboration, and potential manipulations to provide a clearer picture of author performance. Their findings highlighted how to account for self-citations without distortion, advancing a more nuanced and transparent citation analysis [19].

In the current study, self-citation rates were examined for both fields and subfields, with rates of 9.89 % and 10.14 % in 2022. Over the career-long period from 1996 to 2022, the self-citation rates for the fields and subfields were 11.59 % and 11.78 %, respectively. Significant variations were observed among fields and subfields when self-citations were included or excluded. For instance, in 2022, among the 20 fields analysed, Clinical medicine ranked first in total-citations but dropped to 12th place when self-citations were excluded. These significant drops in rankings highlights the profound impact of self-citation on the perceived research impact and visibility within specific academic domains.

When examining the high rates of self-citation observed in certain fields, it is important to consider the role of extensive co-authorship and mega-science collaborations. Changa et al. showed that in fields such as nuclear and particle physics, extensive co-authorship networks significantly affect both total citation and self-citation practices, leading to higher self-citation rates owing to the collaborative nature of research in these areas [20]. Collaborative research often involves a larger number of authors than non-collaborative efforts, naturally increasing the frequency of self-citations, as more contributors may reference their previous works. This effect can act as an ‘amplifier’ of the perceived impact. However, it is crucial to distinguish this phenomenon from instances in which self-citations are strategically employed to artificially enhance citation metrics. Van Raan et al. indicated that while elevated self-citation rates in international collaborations may be prevalent, they do not necessarily serve as ‘impact amplifiers’ in a misleading manner [21].

Self-citations are appropriate when used to credit one's previous contributions or to build upon established research, thereby preventing issues such as self-plagiarism. However, they become problematic if employed solely to artificially enhance citation metrics without contributing meaningful content to the new work. This misuse can distort the perceived significance of publications and journals [22]. Illegitimate self-citations can be direct or indirect. Direct self-citation occurs when authors cite their own previous work, whereas indirect self-citation involves manipulative practices such as coercion by advisors or collusion among scholars to cross-cite each other's work [23]. These indirect methods are challenging to detect using large-scale bibliometric analyses. Self-citation can be defined in two ways: ‘author-based’, where a citation is considered self-citation if the citing and cited papers share at least one author, and ‘publication-based’, where it is defined if any co-author of the citing paper is also a co-author of the cited paper. The latter can sometimes misrepresent the true self-citation behaviour [23]. Self-citation rates are influenced by several factors, including the number of citable papers, number of authors, diversity of references, and presence of international collaborators. Simple analyses often fail to reveal the true impact of incentive schemes on self-citation behaviours. In a study by Abramo et al., Italian professors showed an 9.5 % average increase in self-citations after the national scientific accreditation incentive, with the greatest increase among assistants and associate professors [24]. However, not all professors exhibited this behaviour, highlighting the need to assess whether such increases are genuine or inflated. While excluding self-citations from metrics may penalise honest researchers, a more effective approach would be for editors and reviewers to monitor and address illegitimate self-citations.

Citations from authors of publications originating from their own institutions are called institutional self-citations. This type of self-citation significantly affects an institution's scientific standing. As a result, Glänzel et al. proposed examining the citation impact with and without self-citations to gain deeper insights into the influence of universities and research institutions [25]. An analysis of scholars' self-citations at the institutional level in the current study revealed interesting findings from 1996 to 2022, the self-citation rate of all institutions was 12.74 %. When examining the differences in rankings between total citations and citations without self-citations, substantial variations in decline were observed across most institutions. The Organisation Européenne pour la Recherche Nucléaire showed the most significant decline in ranking variation. It was initially ranked 239th in terms of total citations; its ranking plummeted to 23898^th^ when self-citations were excluded, marking a decline change of 23659. Similarly, the Max-Planck-Institut für Gravitationsphysik (Albert-Einstein-Institut) exhibited the second-highest decline in ranking variation. Initially ranked 1075th in terms of total citations, its ranking dropped to 23972^nd^ when self-citations were excluded, with a decline change of 22897. This notable variation in self-citation suggests a heavy reliance on self-citation practices to support the perceived quality of research output. Moreover, insights obtained from a literature review indicate a negative correlation between performance and self-citation rates [26].

## 5 Conclusion

The findings suggest that the rankings of countries, institutes, fields, and subfields differ significantly when self-citation is excluded, underscoring the critical importance of accounting for excluding self-citation in ranking assessments because a dramatic change in top 2 % of researcher rankings was observed when self-citation was excluded. Additionally, specific fields in which self-citation may increase owing to multi-authorship should be given special attention.

## CRediT authorship contribution statement

**Berun A. Abdalla:** Writing – review & editing, Writing – original draft, Visualization, Validation, Investigation, Data curation. **Ayman M. Mustafa:** Writing – review & editing, Writing – original draft, Visualization, Validation, Software, Resources, Conceptualization. **Fattah H. Fattah:** Writing – review & editing, Visualization, Investigation, Formal analysis, Data curation. **Fahmi H. Kakamad:** Writing – review & editing, Visualization, Validation, Methodology, Conceptualization. **Sami S. Omar:** Writing – review & editing, Validation, Resources, Methodology, Investigation. **Ameer M. Salih:** Writing – review & editing, Visualization, Validation, Software, Formal analysis, Data curation, Conceptualization. **Aso S. Muhialdeen:** Writing – review & editing, Validation, Software, Resources, Methodology, Data curation. **Jaafar Omer Ahmed:** Writing – review & editing, Validation, Software, Resources, Data curation, Conceptualization. **Rawa Bapir:** Writing – review & editing, Visualization, Resources, Formal analysis, Data curation, Conceptualization. **Shvan H. Mohammed:** Writing – review & editing, Validation, Methodology, Investigation, Conceptualization. **Karokh K. Mohammed:** Writing – review & editing, Software, Methodology, Investigation, Data curation, Conceptualization. **Hiwa O. Baba:** Writing – review & editing, Supervision, Resources, Methodology, Formal analysis, Conceptualization. **Sasan M. Ahmed:** Writing – review & editing, Visualization, Validation, Methodology, Formal analysis, Data curation, Conceptualization. **Shevan M. Mustafa:** Writing – review & editing, Visualization, Validation, Investigation, Data curation, Conceptualization. **Kayhan A. Najar:** Writing – review & editing, Visualization, Validation, Resources, Methodology, Investigation, Conceptualization.

## Ethics approval and consent to participate

Not applicable.

## Availability of data and material

All data supporting the findings of this study are included in the article and supplementary materials.

## List of abbreviations

None.

## Funding

This research did not receive any specific funding.

## Declaration of competing interest

The authors declare that they have no known competing financial interests or personal relationships that could have appeared to influence the work reported in this paper.

## References

[1] Shehatta I., Al-Rubaish A.M. (2019). Impact of country self-citations on bibliometric indicators and ranking of most productive countries. Scientometrics.

[2] Sergio Della S., Brooks J. (2008). Multi-authors' self-citation: a further impact factor bias?. Cortex.

[3] Bai X., Liu H., Zhang F., Ning Z., Kong X., Lee I., Xia F. (2017). An overview on evaluating and predicting scholarly article impact. Information.

[4] Li H., Liu W. (2020). Same but different: self-citations identified through Scopus and Web of science core collection. Scientometrics.

[5] Gul S., Shah T.A., Shafiq H. (2017). The prevalence of synchronous self-citation practices at the institutional level. Malays. J. Libr. Inf. Sci..

[6] Yaminfirooz M., Tirgar A. (2019). Self-citation in Iran in comparison with other countries. Acta Inf. Med..

[7] Hyland K. (2003). Self‐citation and self‐reference: credibility and promotion in academic publication. J. Am. Soc. Inf. Sci. Technol..

[8] Pandita R., Singh S. (2015). Impact of self-citations on impact factor: a study across disciplines, countries and continents. Journal of Information Science Theory and Practice.

[9] Ioannidis J.P.A. (2023). October 2023 data-update for “Updated science-wide author databases of standardized citation indicators.”. Elsevier Data Rep..

[10] (2023). World Bank list of economies. https://www.jspn.or.jp/uploads/uploads/files/english/120th_World_Bank_list_of_economies.pdf.

[11] Pandita R., Singh S. (2017). Self-citations, a trend prevalent across subject disciplines at the global level: an overview. Collect. Build.

[12] Fowler J., Aksnes D. (2007). Does self-citation pay?. Scientometrics.

[13] Masic I. (2014). Plagiarism in scientific research and publications and how to prevent it. Mater Socio-méd..

[14] Bardeesi A.M., Jamjoom A.A., Sharab M.A., Jamjoom A.B. (2021). Impact of country self-citation on the ranking of the top 50 countries in clinical neurology. Eneurologicalsci.

[15] Abdullah H.O., Abdalla B.A., Kakamad F.H. (2024). Predatory publishing lists: a review on the ongoing battle against fraudulent actions. Barw Med J.

[16] Baccini A., Petrovich E. (2023). A global exploratory comparison of country self-citations 1996-2019. PLoS One.

[17] Baccini A., De Nicolao G., Petrovich E. (2019). Citation gaming induced by bibliometric evaluation: a country-level comparative analysis. PLoS One.

[18] Bardeesi A.M., Jamjoom A.A., Algahtani A., Jamjoom A. (2021). The impact of country self-citation rate among medical specialties in Saudi Arabia. Cureus.

[19] Kacem A., Flatt J.W., Mayr P. (2020). Tracking self-citations in academic publishing. Scientometrics.

[20] Changa Y.W., Huang M.H., Hyperauthorship M.J. Chiu (2019). A comparative study of genetics and high-energy physics research. Malays. J. Libr. Inf. Sci..

[21] Van Raan A.F. (1998). The influence of international collaboration on the impact of research results: some simple mathematical considerations concerning the role of self-citations. Scientometrics.

[22] Pichappan P., Sarasvady S. (2002). The other side of the coin: the intricacies of author self-citations. Scientometrics.

[23] Ioannidis J.P.A. (2015). A generalized view of self-citation: direct, co-author, collaborative, and coercive induced self-citation. J. Psychosom. Res..

[24] Abramo G., D'Angelo C.A., Grilli L. (2021). The effects of citation-based research evaluation schemes on self-citation behavior. J Informet.

[25] Glänzel W., Debackere K., Thijs B., Schubert A. (2006). A concise review on the role of author self-citations in information science, bibliometrics and science policy. Scientometrics.

[26] Raan A.F. (2008). Bibliometric statistical properties of the 100 largest European research universities: prevalent scaling rules in the science system. J Am Soc Info Sci Tech.

